# The glycolytic enzyme PFKFB3 alleviates DNA damage and chondrocyte senescence in osteoarthritis

**DOI:** 10.1038/s41420-025-02903-0

**Published:** 2025-12-08

**Authors:** Bo Liu, Chenzhong Wang, Ziyu Weng, Yi Yang, Yi Shi, Chi Zhang

**Affiliations:** 1 Department of Orthopedic Surgery, Zhongshan Hospital, Fudan University, Shanghai, China; 2 Department of Kidney Transplantation, Zhongshan Hospital, Fudan University, Shanghai, China; 3 Shanghai Key Laboratory of Organ Transplantation, Zhongshan Hospital, Fudan University, Shanghai, China

**Keywords:** Senescence, Diseases, Mechanisms of disease, Chronic inflammation

## Abstract

Chondrocyte senescence is a key driver of osteoarthritis (OA) progression. This study examined the role of the glycolytic enzyme PFKFB3 in regulating chondrocyte senescence during OA. Using a destabilization of the medial meniscus (DMM) mouse model, we found that PFKFB3 expression was reduced in human and mouse OA cartilage and in hydrogen peroxide–treated chondrocytes. PFKFB3 knockdown or overexpression in primary chondrocytes was achieved through RNA interference or lentiviral delivery, followed by RNA sequencing and molecular analyses. PFKFB3 loss impaired DNA damage repair, activated NF-κB signaling, elevated pro-inflammatory cytokines, and promoted chondrocyte senescence, whereas PFKFB3 overexpression enhanced DNA repair and alleviated OA severity. Pharmacologic inhibition of NF-κB reduced inflammatory and senescent phenotypes in PFKFB3-deficient chondrocytes. These findings indicate that PFKFB3 regulates chondrocyte senescence via NF-κB signaling and DNA damage responses, suggesting PFKFB3 as a potential therapeutic target for OA.

## Introduction

Osteoarthritis (OA), a prevalent joint disease, is characterized by progressive degeneration of articular cartilage [1, 2]. Risk factors for OA include mechanical loading, hereditary predisposition, and cellular senescence [3–5]. Senescent chondrocytes in osteoarthritic cartilage exhibit increased senescence-associated secretory phenotypes (SASPs), DNA damage, and blunted repair [6]. Removal of senescent chondrocytes attenuates OA progression, reduces pain, and promotes cartilage regeneration [7–9]. Reduced expression of anti-aging proteins, such as interferon regulatory factor 1 and sirtuin 6, accelerates chondrocyte senescence in OA [10, 11]. Notably, the absence of regulated in development and DNA damage response 1 (REDD1), a crucial protein activated during DNA damage, impairs autophagy and mitochondrial biogenesis in articular chondrocytes and exacerbates cartilage degradation [12, 13].

The glycolytic enzyme 6-phosphofructo-2-kinase/fructose-2,6-bisphosphatase (PFKFB) family comprises four isozymes (PFKFB1-4). Despite their high sequence homology (85%) in core catalytic domains, these isozymes exhibit distinct tissue expression profiles and responses to extracellular stimuli [14]. Among these, PFKFB3 exhibits the highest kinase-to-phosphatase activity ratio, enabling it to sustain high glycolytic rates, since PFKFB3 overexpression significantly enhances glycolytic function in OA cartilage explants and human chondrocytes. Furthermore, PFKFB3 overexpression counteracts the suppression of glycolysis induced by inflammatory cytokines such as TNF-α and IL-1β [15]. These findings establish PFKFB3 as an important regulator of chondrocyte metabolism under pathophysiological conditions.

Apart from its well-studied role in glycolysis, PFKFB3 has also been reported to translocate to the nucleus and regulate DNA repair. In response to oxidative stress, PFKFB3 moves to DNA damage sites in human umbilical vein endothelial cells, promoting DNA repair through interactions with the MRN-ATM pathway [16]. In cancer cells, PFKFB3 rapidly translocates to ionizing radiation (IR)-induced nuclear foci via an MRN-ATM-γH2AX-MDC1-dependent mechanism, facilitating the recruitment of homologous recombination (HR) proteins and maintaining HR activity [17]. Thus, it indicates that PFKFB3 is a pleiotropic regulator. However, whether PFKFB3 plays a role in regulating DNA damage and senescence in osteoarthritic chondrocytes remains to be investigated.

The present study aimed to determine the presence of PFKFB3 in osteoarthritis and investigate its role in DNA damage and chondrocyte senescence in OA cartilage. By employing RNA interference (RNAi) to inhibit PFKFB3 expression and adeno-associated virus (AAV) to induce its overexpression, we hypothesized that PFKFB3 exerts protective effects on chondrocytes by mitigating senescence and DNA damage.

## Results

### PFKFB3 expression is decreased in osteoarthritic cartilage

By analyzing bulk RNA sequencing datasets (GSE241794 and GSE114007) and single-cell RNA sequencing (scRNA-seq) data (GSE104782), PFKFB3 was identified as the predominant isoform in the PFKFB family in cartilage tissue (Supplementary Fig. 1a–c). PFKFB3 mRNA expression was significantly downregulated in osteoarthritic (OA) cartilage from both human and rat models (Supplementary Fig. 1b, c).

**Fig. 1:**
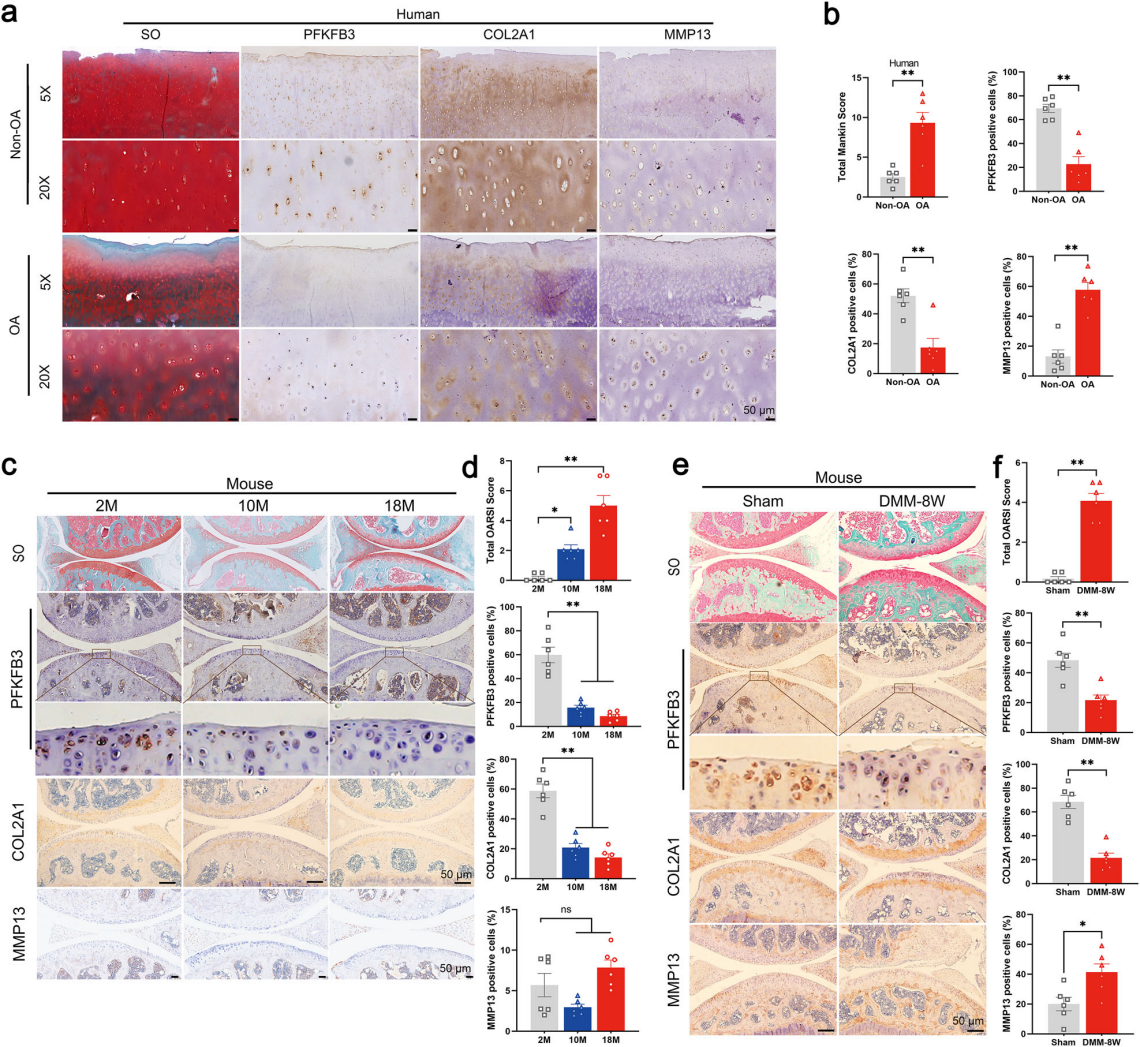
PFKFB3 plays an important role in the development of osteoarthritis (OA). **a** Safranin O-Fast Green (SO&FG) staining (Left) and immunohistochemical (IHC) staining of PFKFB3, COL2A1, and MMP13 in human normal and OA cartilage. **b** Mankin scores and quantification of PFKFB3, COL2A1, and MMP13 signals in human non-OA and OA cartilage. Data presented as means ± s.e.m., n = 6, unpaired Student’s *t*-test. **c** SO&FG staining and IHC staining in the cartilage of 2-month-old, 10-month-old, and 18-month-old mice. **d** Osteoarthritis Research Society International (OARSI) scores and quantification of PFKFB3, COL2A1, and ACAN expression in the cartilage of aged mice. Data presented as means ± s.e.m., n = 6, one-way ANOVA with Tukey’s comparisons. **e** SO&FG staining and IHC staining in the cartilage of mice subjected to 8 weeks after DMM surgery. **f** OARSI scores and quantification of PFKFB3, COL2A1, and MMP13 signals in the cartilage of mice subjected to DMM surgery. Data presented as means ± s.e.m., n = 6, unpaired Student’s *t*-test. *P < 0.05, **P < 0.01. ns not significant.

Twelve human cartilage samples were collected in the present study (Supplementary Table 1). Compared with non-OA samples, OA cartilage had a reduced expression of type II collagen (COL2A1), a key extracellular matrix (ECM) protein, and increased levels of matrix metalloproteinase-13 (MMP13), a primary matrix-degrading enzyme for COL2A1 (Fig. 1a, b). Cartilage degradation in OA samples was further supported by diminished Safranin O–Fast Green (SO&FG) staining (Fig. 1a, b). In line with these findings, PFKFB3 expression was significantly reduced in OA cartilage compared with non-OA controls (Fig. 1a, b).

In both surgical destabilization of the medial meniscus (DMM)-induced and aging-associated OA mouse models, SO&FG staining and OARSI scoring confirmed cartilage degeneration (Fig. 1c–f). Compared to their young counterparts, aged mice showed decreased COL2A1 expression in cartilage tissues (Fig. 1c, d). In contrast, no significant difference in MMP13 expression was detected (Fig. 1c, d). Cartilage from mice 8 weeks post-DMM surgery exhibited reduced COL2A1 and elevated MMP13 expression (Fig. 1e, f). Notably, PFKFB3 expression was markedly decreased in both OA models (Fig. 1c–f).

### PFKFB3 regulates DNA damage and senescence in chondrocytes

**Fig. 2:**
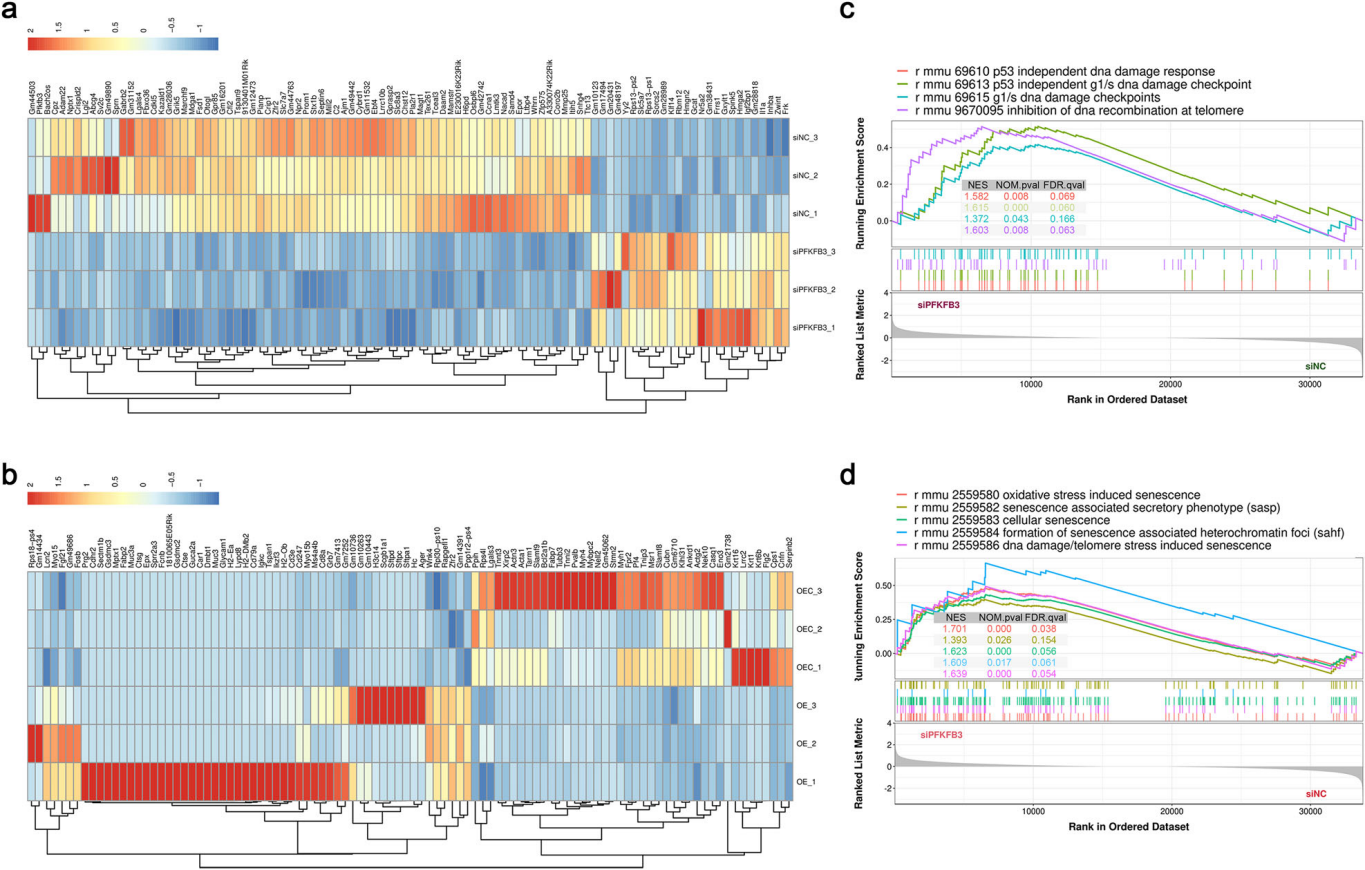
RNA-seq analysis for primary chondrocytes with PFKFB3 knockdown and overexpression. **a** Heatmap showing the top 100 differentially expressed genes (DEGs) in primary mouse chondrocytes transfected with siPFKFB3. **b** Heatmap showing the top 100 DEGs in primary mouse chondrocytes transfected with lenti-PFKFB3. **c** Gene Set Enrichment Analysis (GSEA) associated with DNA damage response and **d** cellular senescence in PFKFB3-knockdown chondrocytes.

**Fig. 3:**
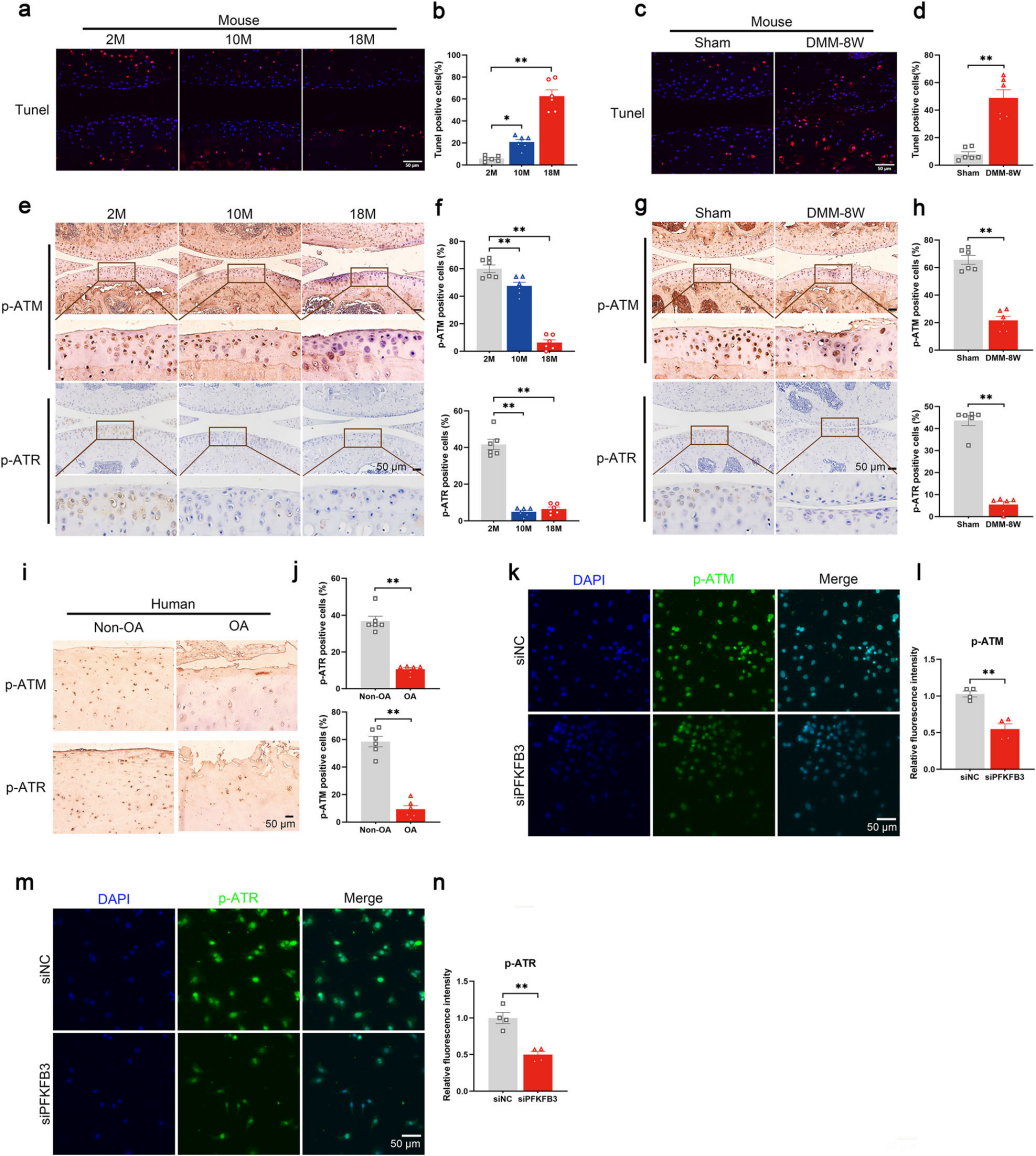
Increased DNA damage and impaired DNA damage repair in human and mouse OA cartilage. **a** Representative images and **b** quantification of terminal deoxynucleotidyl transferase dUTP nick end labeling (TUNEL) staining in the cartilage of 2-month-old, 10-month-old, and 18-month-old mice. Data presented as means ± s.e.m., n = 6, one-way ANOVA with Tukey’s comparisons. **c** Representative TUNEL staining and **d** quantification of TUNEL signals in the cartilage of mice, 8 weeks after the DMM surgery. Data presented as means ± s.e.m., n = 6, unpaired Student’s *t*-test. **e** Immunohistochemical (IHC) staining and **f** quantification of p-ATM (upper panel) and p-ATR (lower panel) in the cartilage of aged mice. Data presented as means ± s.e.m., n = 6, one-way ANOVA with Tukey’s comparisons. **g** IHC staining and **h** quantification of p-ATM (upper panel) and p-ATR (lower panel) in the cartilage of mice subjected to DMM surgery. Data presented as means ± s.e.m., n = 6, unpaired Student’s *t*-test. **i** IHC staining and **j** quantification of p-ATM (upper panel) and p-ATR (lower panel) in human normal and OA cartilage. Data presented as means ± s.e.m., n = 6, unpaired Student’s t-test. **k** IF staining and **l** quantification of p-ATM in chondrocytes transfected with siPFKFB3. Data presented as means ± s.e.m., n = 4, paired Student’s *t*-test. **m** IF staining and (**n**) quantification of p-ATR in chondrocytes transfected with siPFKFB3. Data presented as means ± s.e.m., n = 4, paired Student’s *t*-test. *P < 0.05, **P < 0.01. ns not significant.

To explore its role in chondrocytes, PFKFB3 expression was modulated using small interfering RNA (siRNA) for knockdown and lentiviral vectors for overexpression, respectively (Fig. 2a, b and Supplementary Fig. 2a–f). Gene Set Enrichment Analysis (GSEA) identified significant enrichment of DNA damage and cellular senescence in both PFKFB3-knockdown and PFKFB3-overexpressed chondrocytes (Fig. 2c, d and Supplementary Fig. 2g, h). These bioinformatics and experimental findings suggest that PFKFB3 is pivotal in regulating DNA damage and senescence in chondrocytes. Increased terminal deoxynucleotidyl transferase dUTP nick end labeling (TUNEL) signals and reduced phosphorylated forms of ataxia telangiectasia mutated (ATM) and ataxia telangiectasia and Rad3-related (ATR), two essential components of the DNA-repair response, confirmed the DNA strand breaks in human and mouse OA cartilage (Fig. 3a–j). Notably, PFKFB3 knockdown reduced p-ATM and p-ATR levels in chondrocytes (Fig. 3k–n).

### PFKFB3 alleviates DNA damage and chondrocyte senescence

**Fig. 4:**
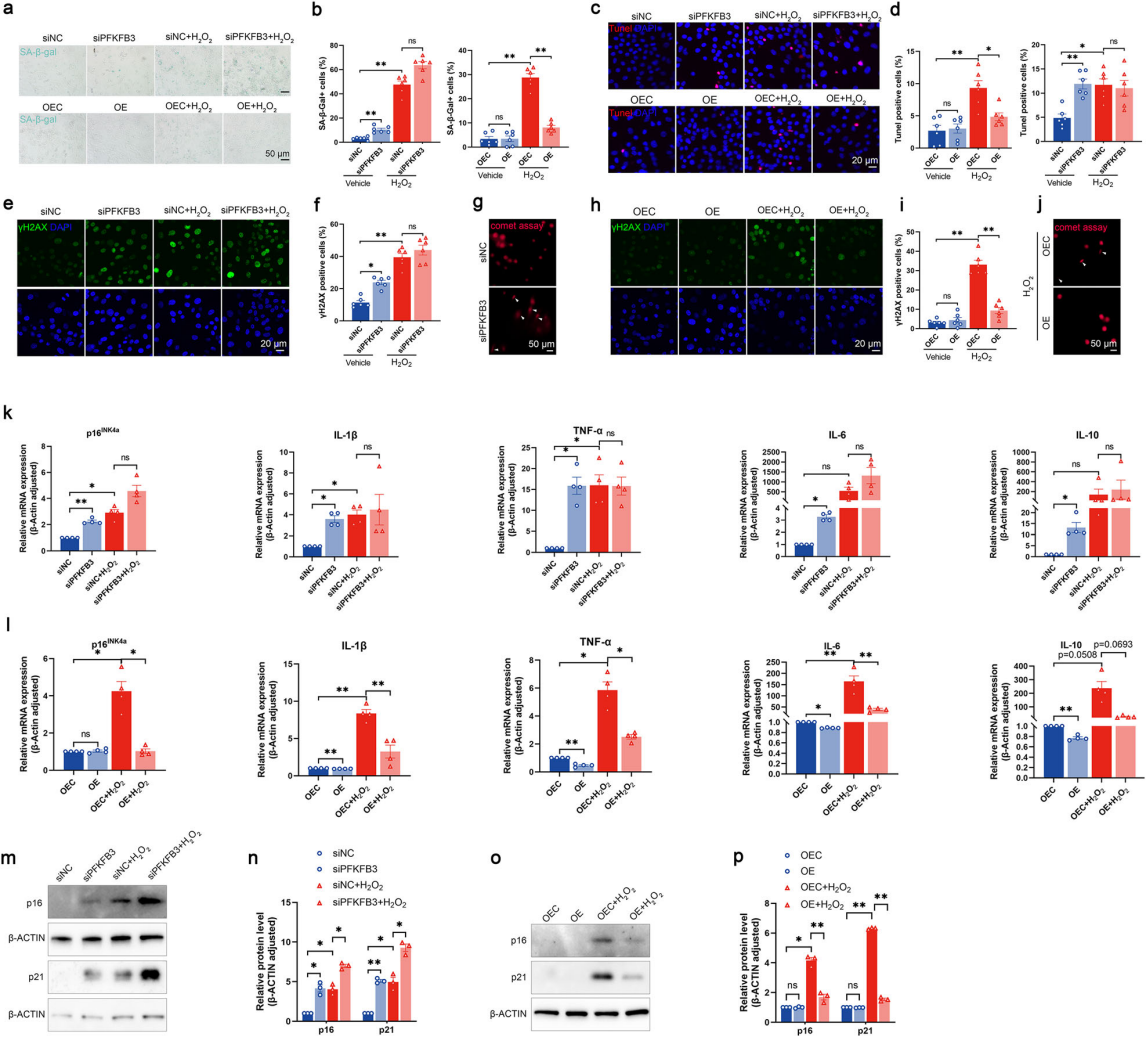
PFKFB3 alleviates DNA damage and cellular senescence. **a** Representative images and **b** quantification of senescence-associated β-galactosidase (SA-β-Gal) staining in H_2_O_2_-stimulated chondrocytes transfected with siPFKFB3 (upper panel) or transfected with lenti-PFKFB3 (lower panel). Data presented as means ± s.e.m., n = 6, one-way ANOVA with Tukey’s multiple comparisons. **c** Representative images and **d** quantification of TUNEL staining in H_2_O_2_-stimulated chondrocytes transfected with siPFKFB3 (upper panel) or lenti-PFKFB3 (lower panel). Data presented as means ± s.e.m., n = 6, one-way ANOVA with Tukey’s multiple comparisons. **e** Immunofluorescence staining and **f** quantification of γH2AX signals in H_2_O_2_-stimulated chondrocytes transfected with siPFKFB3. Data presented as means ± s.e.m., n = 6, one-way ANOVA with Tukey’s multiple comparisons. **g** Representative images of comet assay in chondrocytes transfected with siPFKFB3. **h** Representative images of IF staining and **i** quantification of γH2AX signals in H_2_O_2_-stimulated chondrocytes transfected with lenti-PFKFB3. Data presented as means ± s.e.m., n = 6, one-way ANOVA with Tukey’s multiple comparisons. **j** Representative images of comet assay in H_2_O_2_-stimulated chondrocytes transfected with lenti-PFKFB3. **k** mRNA expression of pro-inflammatory factors, including p16^INK4a^, TNF-α, IL-1β, IL-6, and IL-10 in H_2_O_2_-stimulated chondrocytes transfected with siPFKFB3. Data presented as means ± s.e.m., n = 4, one-way ANOVA with Tukey’s multiple comparisons. **l** mRNA expression of p16^INK4a^, TNF-α, IL-1β, IL-6, and IL-10 in H_2_O_2_-stimulated chondrocytes transfected with lenti-PFKFB3. Data presented as means ± s.e.m., n = 4, one-way ANOVA with Tukey’s multiple comparisons. **m** Representative blots and **n** densitometric quantification of p16^INK4a^ and p21 in H_2_O_2_-stimulated chondrocytes transfected with siPFKFB3. Data presented as means ± s.e.m., n = 3, one-way ANOVA with Tukey’s multiple comparisons. **o** Representative blots and **p** densitometric quantification of p16^INK4a^ and p21 in H_2_O_2_-stimulated cells transfected with lenti-PFKFB3. Data presented as means ± s.e.m., n = 3, one-way ANOVA with Tukey’s multiple comparisons. *P < 0.05, **P < 0.01. ns not significant.

To mimic aging-associated senescent conditions in vitro, hydrogen peroxide (H_2_O_2_) was used in the present study [18]. As the concentration of H₂O₂ increased, cell viability was significantly reduced when hydrogen peroxide reached 400 μM (Supplementary Fig. 3a). At lower concentrations (25–200 μM), H₂O₂ stimulation significantly upregulated the protein levels of p16^INK4a^, p21, and MMP13, and downregulated the protein level of COL2A1 in a dose-dependent manner (Supplementary Fig. 3b, c). Therefore, 200 μM was used in the present study. Immunofluorescence and cellular-fractionation assays revealed that PFKFB3 was mainly distributed in the cytoplasm of mouse chondrocytes, and its expression was significantly reduced in the presence of H₂O₂ (Supplementary Fig. 3d–g). Furthermore, H₂O₂ exposure markedly induced cellular senescence, apoptosis, and DNA damage in chondrocytes, as evidenced by increased senescence-associated β-galactosidase (SA-β-Gal) staining, TUNEL signals, and enhanced DNA damage marker γH2AX foci formation (Fig. 4a–j).

PFKFB3 knockdown induced cellular senescence, apoptosis, and DNA damage under basal conditions, but not in cells stimulated with H₂O₂ (Fig. 4a–g; Supplementary Fig. 4). In contrast, overexpressing PFKFB3 significantly downregulated H₂O₂-induced senescence and apoptosis, and reduced DNA breaks and damage (Fig. 4a–d, h–j and Supplementary Fig. 4). Furthermore, PFKFB3 knockdown significantly increased mRNA levels of SASP molecules under basal conditions, including p16^INK4a^, interleukin (IL)-1β, tumor necrosis factor-alpha (TNF-α), IL-6, and IL-10, but not in cells exposed to H_2_O_2_ stimulation (Fig. 4k). In line, overexpressing PFKFB3 markedly reduced H_2_O_2_-induced SASPs upregulation, but had negligible effects under basal conditions (Fig. 4l). Of note, in the presence of H₂O₂, siPFKFB3 increased the protein expression of p16^INK4a^ and p21 (Fig. 4m, n). Overexpressing PFKFB3 reduced the H₂O₂-induced p16^INK4a^ and p21 upregulation (Fig. 4o, p).

Ionizing radiation (IR) and etoposide stimulation, inducers of cellular senescence and DNA damage, were also used [19–21]. Both stimulations significantly induced chondrocyte senescence and DNA damage (Supplementary Fig. 5). PFKFB3 overexpression significantly alleviated IR- and etoposide-induced cellular senescence and DNA damage (Supplementary Fig. 5).

### Involvement of the nuclear factor-κB (NF-κB) pathway in PFKFB3-mediated reduction of senescence

**Fig. 5:**
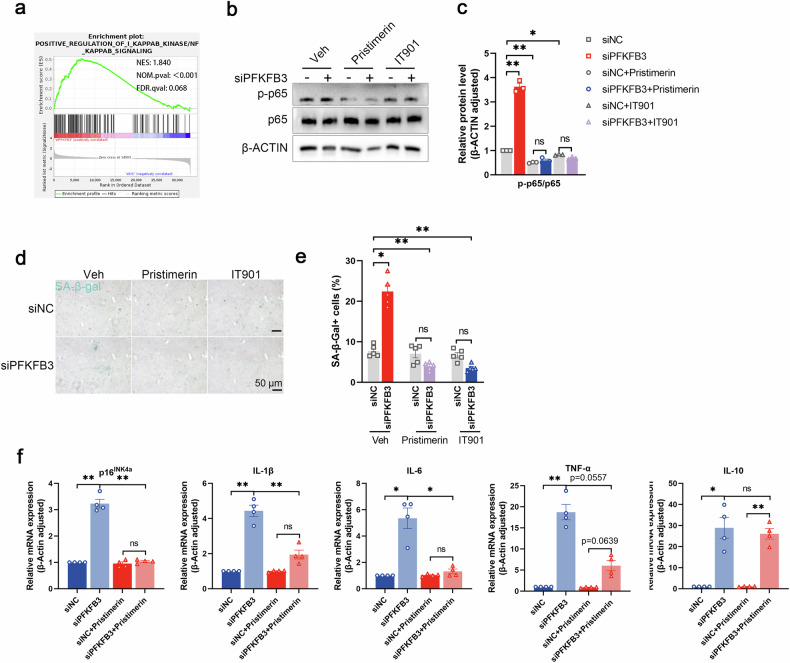
The NF-κB pathway mediates the senescence-inhibitory effect in PFKFB3-knockdown chondrocytes. **a** The NF-κB signaling pathways enriched in PFKFB3-knockdown chondrocytes in Gene Set Enrichment Analysis. **b** Representative blots and **c** densitometric quantification of p65 and p-p65 in cultured cells transfected with siPFKFB3 in the presence of NF-κB inhibitors, pristimerin (500 nM) and IT901 (150 nM). Data presented as means ± s.e.m., n = 3, one-way ANOVA with Tukey’s multiple comparisons. **d** Senescence-associated β-galactosidase (SA-β-Gal) staining and **e** quantification of SA-β-Gal-positive chondrocytes transfected with siPFKFB3 in the presence of pristimerin and IT901. Data presented as means ± s.e.m., n = 5, one-way ANOVA with Tukey’s multiple comparisons. **f** mRNA expression of pro-inflammatory factors in cultured chondrocytes transfected with siPFKFB3 in the presence of pristimerin. Data presented as means ± s.e.m., n = 4, one-way ANOVA with Tukey’s multiple comparisons. *P < 0.05, **P < 0.01. ns not significant.

To investigate potential signaling pathways involved in PFKFB3-mediated senescence regulation, GSEA revealed that the positive regulation of NF-κB signaling was enriched (Fig. 5a). To further explore the participation of NF-κB in PFKFB3-mediated chondrocyte senescence, two pharmacological inhibitors, pristimerin (500 nM) and IT901 (150 nM), were used. PFKFB3 knockdown increased p65 phosphorylation, but not the total p65 protein. PFKFB3 knockdown did not affect IKKα/β protein (Fig. 5b, c and Supplementary Fig. 6). NF-κB inhibitors reduced the SA-β-Gal signals in chondrocytes (Fig. 5d, e) and decreased mRNA levels of SASP molecules (Fig. 5f).

Co-immunoprecipitation coupled with mass spectrometry revealed no physical interactions between PFKFB3 and NF-κB-related proteins, including NF-κB subunits (p65 and p50) and regulatory kinases (IKKα, IKKβ, or IKKγ), in 293 T cells overexpressing Flag-tagged PFKFB3 (data not shown). This observation agreed with the lack of clearly detectable nuclear PFKFB3 in both immunofluorescence and immunoblotting experiments (Supplementary Fig. 3f).

### PFKFB3 alleviates post-traumatic osteoarthritis in vivo

**Fig. 6:**
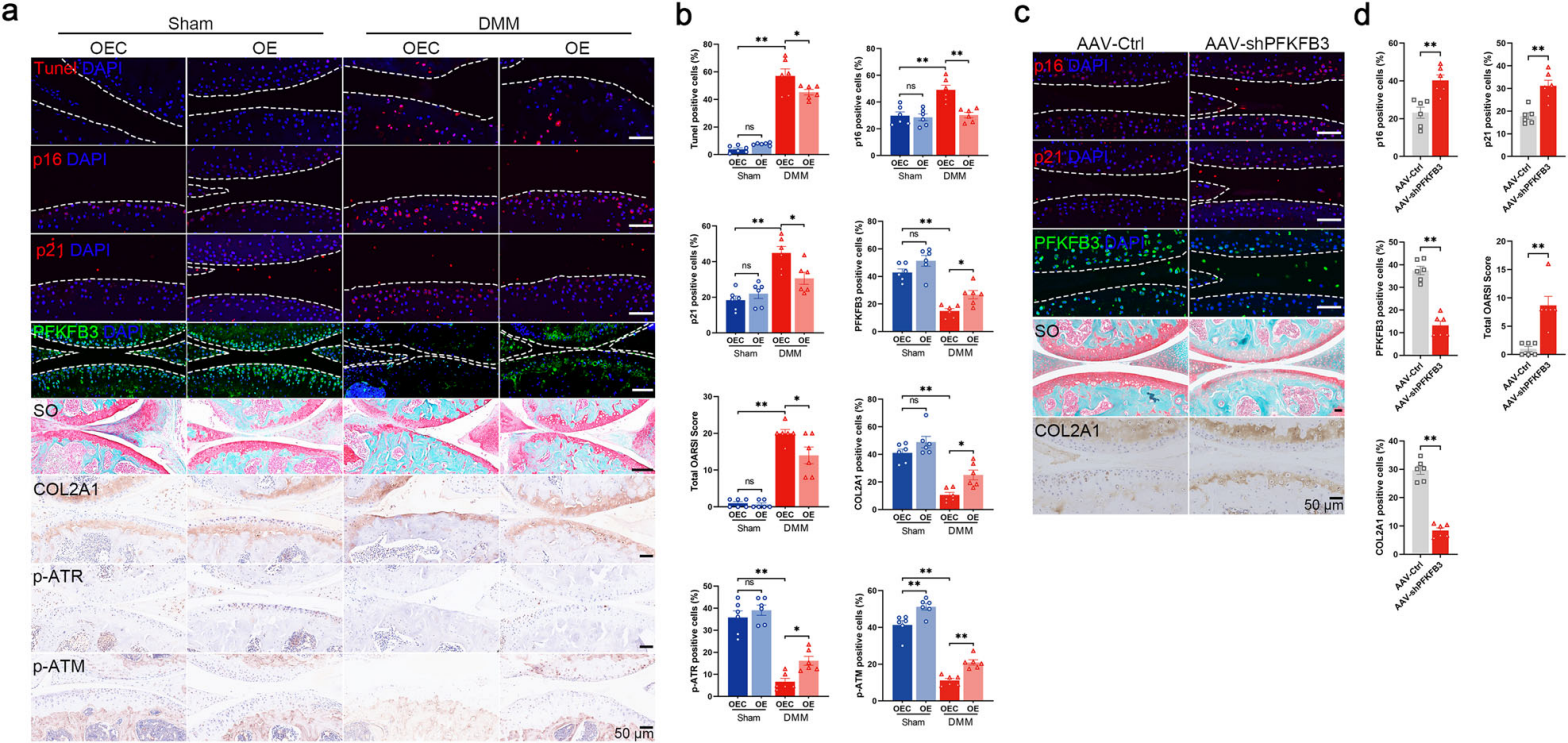
PFKFB3 protects cartilage from post-traumatic osteoarthritis in vivo. **a** Safranin O-Fast Green (SO&FG) staining, immunofluorescence (IF) staining of PFKFB3, p16^INK4a^, and p21, immunohistochemical (IHC) staining of COL2A1, MMP13, p-ATM, and p-ATR in the cartilage of mice subjected to 8 weeks post-DMM surgery and administered with AAV-PFKFB3 via intra-articular injection. **b** Osteoarthritis Research Society International (OARSI) scores and quantification of TUNEL, PFKFB3, COL2A1, p-ATM, and p-ATR signals in the cartilage of mice subjected to DMM surgery and injected with AAV-PFKFB3. Data presented as means ± s.e.m., n = 6, one-way ANOVA with Tukey’s multiple comparisons. **c** SO&FG staining, IF staining of p16^INK4a^, p21, and PFKFB3, and IHC staining of COL2A1 in the cartilage of mice administered with AAV-shPFKFB3 via intra-articular injection. **d** OARSI scores and quantification of p16^INK4a^, p21, PFKFB3, and COL2A1 signals in the cartilage of mice injected with AAV-shPFKFB3. Data presented as means ± s.e.m., n = 6, unpaired Student’s *t*-test. *P < 0.05, **P < 0.01. ns not significant.

To confirm the protective effects of PFKFB3 on osteoarthritic cartilage, adeno-associated virus serotype 9 (AAV) vectors expressing PFKFB3 (AAV-PFKFB3) and a negative control (AAV-Ctrl) were administered via intra-articular injection into mice undergoing DMM surgery. AAV-PFKFB3 treatment significantly increased PFKFB3 expression in the cartilage of both Sham and DMM groups compared to the AAV-Ctrl group (Fig. 6a, b). SO&FG staining demonstrated severe cartilage damage in DMM mice, which was partially ameliorated in AAV-PFKFB3-treated mice 8 weeks after the surgery (Fig. 6a, b). The protective effects of AAV-PFKFB3 treatment were also observed as reduced TUNEL staining (Fig. 6a, b), increased levels of p-ATM and p-ATR, and upregulated COL2A1 expression (Fig. 6a, b). Conversely, intra-articular injection of AAV-shPFKFB3 resulted in severe articular cartilage destruction, shown as reduced expression of COL2A1 and promoted chondrocyte senescence and apoptosis compared with the control group (Fig. 6c, d).

## Discussion

The present study reveals a novel role for PFKFB3 in regulating DNA damage and cellular senescence in chondrocytes during osteoarthritis. Reduced expression of the glycolytic enzyme PFKFB3 activates NF-κB signaling, promotes the release of SASP molecules, and impairs DNA repair in osteoarthritic cells.

Chondrocytes produce ATP via glycolysis [22–25], as evidenced by upregulated glycolytic enzymes pyruvate kinase M2 and lactate dehydrogenase A in mice with osteoarthritis and senescent chondrocytes [25]. Thus, the reduced PFKFB3 expression in osteoarthritic chondrocytes in the present study implies that PFKFB3 plays protective roles beyond glycolysis. The blunted glycolysis in the previous publication [15], shown as reduced lactate levels in cultured chondrocytes exposed to TNFα and IL-1β, is probably due to the use of C20/A4 chondrocytes. Therefore, primary chondrocytes are recommended for in vitro studies.

In the present study, cellular senescence observed in PFKFB3-knockdown chondrocytes and PFKFB3-knockdown mice confirms the participation of cellular senescence in osteoarthritis progression. Of importance, the PFKFB3 protein and phosphorylation of ATM and ATR kinases showed parallel changes in human and mouse cartilage. These findings imply that PFKFB3 is a crucial player in senescence [16, 17], since DNA damage and impaired DNA repair are critical drivers of cellular senescence [26, 27].

The NF-κB pathway, a well-known regulator of inflammation and immunity [28, 29], is activated upon PFKFB3 deletion in ovarian cancer [30] and proximal tubular cells challenged with ischemia-reperfusion injury [31]. PFKFB3 knockdown enhances NF-κB expression via histone lactylation at gene promoters in mouse kidney models subjected to ischemia-reperfusion injury [31]. The data showing increased expression of SASP molecules in PFKFB3-knockdown chondrocytes under quiescent, but not stimulated conditions, indicate that PFKFB3 has obligatory protection on chondrocytes [32, 33].

**Fig. 7:**
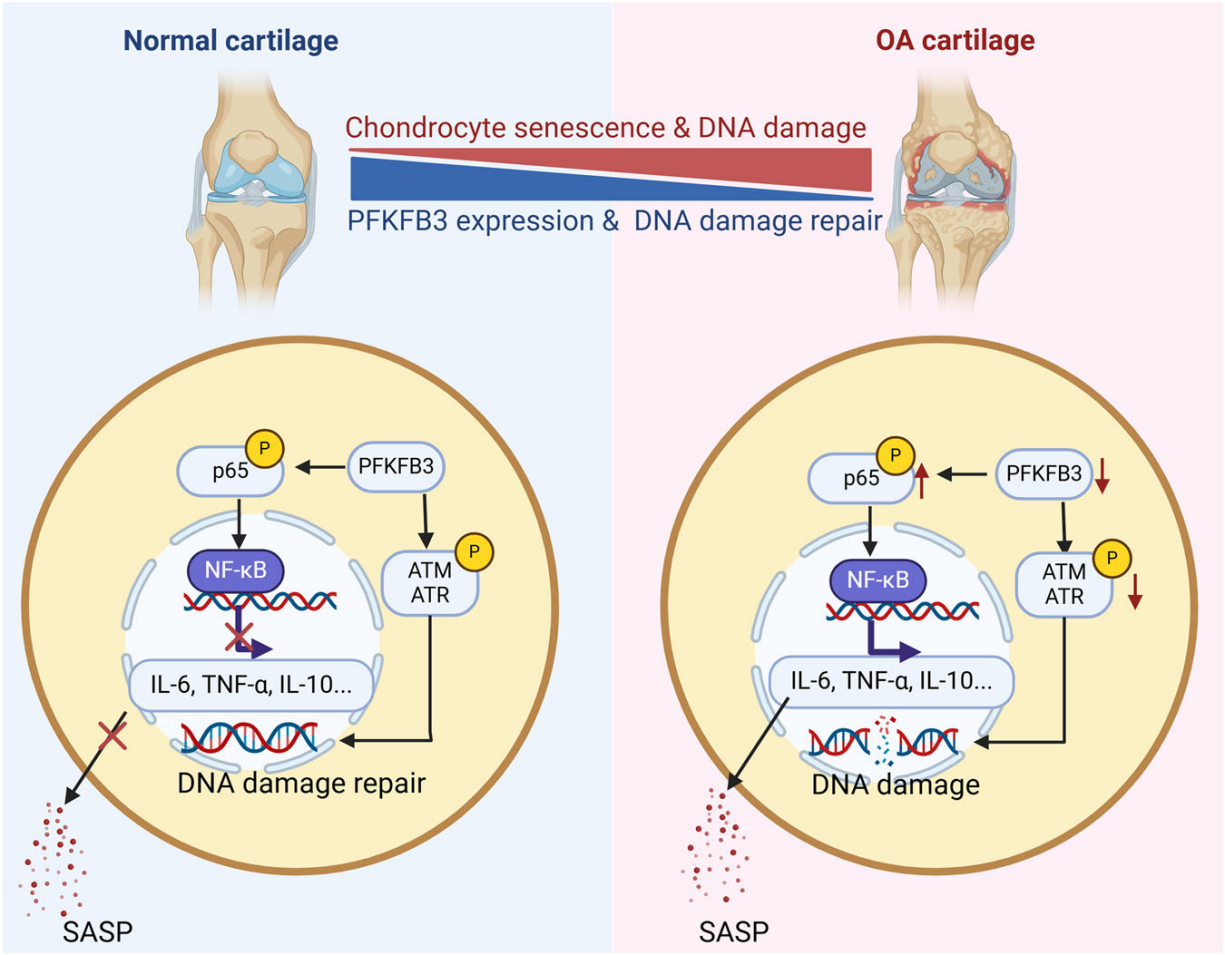
PFKFB3 alleviates chondrocyte DNA damage and senescence in osteoarthritis. PFKFB3 is critical in cartilage under physiological conditions by inhibiting NF-κB activation and promoting DNA repair. In the progression of osteoarthritis, reduced PFKFB3 expression activates the NF-κB signaling pathway and impairs DNA repair, leading to increased levels of proinflammatory factors, as well as accumulated DNA damage and chondrocyte senescence.

ATM/ATR and NF-κB activation occur in the nucleus. The nuclear PFKFB3 regulates mitosis and DNA repair in malignant hepatocytes [34, 35] and endothelial cells [36]. The distinct translocation of PFKFB3 is probably due to the cell-type specificity of its function. The absence of detectable nuclear PFKFB3 and lack of physical interactions with ATM/ATR kinase or NF-κB proteins suggest an indirect regulatory mechanism in the present study. Notably, PFKFB3 positively regulates ATM/ATR phosphorylation and negatively regulates NF-κB p65 phosphorylation, supporting the statement that PFKFB3 has a higher capacity to regulate kinase phosphorylation. Thus, further investigation into PFKFB3-mediated phosphorylation is warranted (Fig. 7).

Glycolysis is enhanced in malignant [37, 38] and immune cells [39–41]. Vascular endothelial cells, despite high oxygen availability, predominantly utilize aerobic glycolysis [42, 43]. Cartilage, located inside the articular cavity and chronically exposed to hypoxia [44], favors glycolysis for ATP generation [44]. PFKFB3 promotes tumor growth and angiogenesis in cancers [38] and confers radioresistance and chemotherapy-resistance in cancer treatments [45, 46]. Downregulating PFKFB3 impedes angiogenesis in diabetic nephropathy [47] and disrupts M2 macrophage polarization of bone-marrow-derived macrophages [48]. In retinal myeloid cells, deleting PFKFB3 prevents macrophages/microglia from differentiating into an angiogenic phenotype in a mouse oxygen-induced proliferative retinopathy model [49]. Deleting PFKFB3 in astrocytes reduces neuronal energy supply and exacerbates neuronal loss in treating spinal cord injury [50].

It is intriguing that the glycolytic enzyme PFKFB3 has an obligatory inhibition on NF-κB signaling. Thus, the upstream signals controlling the basal PFKFB3 expression are of great importance. Hypoxia-inducible factor-1α binds to the PFKFB3 promoter and upregulates PFKFB3 expression in hepatocellular carcinoma cells [34, 35]. Sirtuin 6, a senescence regulatory factor, suppresses [51] or promotes [52] glycolysis by regulating PFKFB3. Interferon regulatory factor 1 prevents senescence by repairing DNA damage [11] or promotes senescence-associated SASP molecules [53]. Laminar blood flow upregulates Kruppel-like factor 2, which represses PFKFB3 expression and decreases glycolysis in vascular endothelial cells [36]. Posttranscriptional and epigenetic mechanisms participate in the regulation of PFKFB3 protein expression as well [54, 55].

Several limitations should be acknowledged in the present study. First, patients in the osteoarthritis (OA) group were older than those in the control group. While this observation supports the notion that OA is an age-associated disease, age-related physiological changes may introduce potential bias into the analysis. To address this concern, both aging-related and DMM-induced mouse models were employed. Future investigations should therefore include larger, age-matched clinical cohorts to further validate these findings. Adeno-associated virus (AAV) has been widely utilized as a gene delivery vehicle in clinical applications owing to its non-pathogenic nature and low immunogenicity [56]. However, individuals with pre-existing anti-AAV antibodies may exhibit reduced therapeutic efficacy. Moreover, AAV vectors have a limited packaging capacity of approximately 4.55 kilobases [57]. In the context of regenerative medicine, cell-free therapies, such as exosomes derived from cartilage stem/progenitor cells, have emerged as promising tools to facilitate knee cartilage repair in animal models and promote the repair of osteoarthritic chondrocytes [58, 59]. Thus, delivering PFKFB3 via exosomes or nanomaterial-based systems may represent a promising therapeutic strategy for OA. Nevertheless, the long-term safety and potential adverse effects of PFKFB3 require thorough evaluation prior to clinical translation. Furthermore, the molecular mechanisms linking PFKFB3 to the activation of NF-κB and ATM/ATR pathways warrant further investigation.

In conclusion, the present study demonstrates that PFKFB3 mitigates DNA damage and cellular senescence in osteoarthritic chondrocytes by promoting DNA repair pathways. Targeting PFKFB3 may offer a novel therapeutic approach for osteoarthritis associated with aging, trauma, or radiotherapy.

## Methods and materials

### Reagents

Hydrogen peroxide (H_2_O_2_, #7722-84-1) was sourced from Sigma-Aldrich (St. Louis, USA). Etoposide was obtained from Beyotime (#SC0173, Shanghai, China). Pristimerin (#HY-N1937) and IT901 (#HY-124179) were purchased from MedChemExpress (Monmouth Junction, NJ, USA).

### Human cartilage

Non-OA cartilage samples were obtained from six individuals with no history of OA. OA cartilage samples were collected from six patients who underwent total knee arthroplasty at Zhongshan Hospital, Fudan University, Shanghai, China. All participants in this experiment were aware of the use of their biological samples, and corresponding informed consent was provided. This study was approved by the Ethics Committee of Zhongshan Hospital (No B2020-151R).

### Intra-articular injections and experimental OA in mice

Recombinant adeno-associated virus serotype 9 (AAV9) carrying either PFKFB3 overexpression (AAV-PFKFB3) or knockdown (AAV-shPFKFB3) constructs, along with negative control particles (AAV-Ctrl), were obtained from GenePharma (Shanghai, China). To overexpress the PFKFB3 protein, AAV-Ctrl and AAV-PFKFB3 (5 × 10¹¹ vg/mL in 10 μL) were administered via intra-articular injection for 2 weeks before the surgery. To delete the PFKFB3 protein, AAV-shCtrl and AAV-shPFKFB3 (8 × 10¹⁰ vg/mL in 10 μL) were administered weekly via intra-articular injection for 8 weeks.

The right knees of 12-week-old male C57BL/6 mice underwent the DMM surgery to establish an OA mouse model [60]. The joint capsule was opened without transection of the meniscotibial ligament in mice in the sham group. Mice were sacrificed 8 weeks after the DMM surgery. The experiments were approved by the Ethics Committee for Animal Research of Zhongshan Hospital (No: 2024-015).

### SO&FG staining, IHC, and IF staining

Human cartilage and mouse knee joint tissues were prepared by Boerfu Biotechnology Co., Ltd. (Wuhan, China). Samples were cut into 5 μm sections for histological examination. In Safranin O-Fast Green (SO&FG) staining, slides were stained with 0.1% Safranin O solution (#TMS-009, Sigma-Aldrich) and then 0.001% Fast Green solution (#F7252, Sigma-Aldrich). The Mankin histological grading system [61] and the Osteoarthritis Research Society International (OARSI) scoring system [62] were employed to assess the severity of human and mouse cartilage damage. For the OARSI guidelines, scores representing the sum of four quadrants (medial and lateral tibial plateau and femoral condyle) were blindly assessed by an experienced investigator. In immunohistochemistry, samples underwent a heat-induced epitope retrieval and inactivation of endogenous peroxidase (Gene Tech, Shanghai, China). After blocking with goat serum for 1 h, primary antibodies against PFKFB3 (Cell Signaling Technology, D7H4Q; dilution 1:100), COL2A1 (Proteintech, 28459-1-AP; dilution 1:400), MMP13 (Proteintech, 18165-1-AP; dilution 1:200), p-ATM (Cell Signaling Technology, #4526; dilution 1:200), p-ATR (Cell Signaling Technology, #30632; dilution 1:200) were incubated overnight at 4 °C. Secondary antibody, goat anti-rabbit (Yeasen, 33101ES60; dilution 1:200), was incubated for 1 h. Images were taken using a Digital Pathology Scanner (Leica, Wetzlar, Germany).

In immunofluorescence, after antigen retrieval, primary antibodies against γH2AX (Abcam, ab181861; dilution 1:100), PFKFB3 (Cell Signaling Technology, D7H4Q; dilution 1:50), p16^INK4a^ (Cell Signaling Technology, #29271; 1:100), p21 (Santa Cruz, sc-397; 1:50), p-ATM (Cell Signaling Technology, #4526; dilution 1:100), p-ATR (Cell Signaling Technology, #30632; dilution 1:100) were incubated overnight. Then, slides were incubated with Alexa Fluor 488-conjugated secondary antibodies (Jackson ImmunoResearch, 111-545-003; dilution 1:500) for 1 h and 4′,6-diamidino-2-phenylindole (DAPI; Beyotime) for another 10 min at room temperature. Images were captured using a confocal laser scanning microscope (Olympus, Tokyo, Japan).

### Small interfering RNA (siRNA) transfection

Small interfering RNAs (siRNAs) were synthesized by GenePharma (Supplementary Table 2). Cells were transfected with 50 nM siRNA for 2 days using Lipofectamine RNAiMAX (#13778150, Invitrogen, Carlsbad, USA), following the manufacturer’s instructions.

### Lentivirus infection of chondrocytes

Lenti-PFKFB3 was purchased from GenePharma. Lenti-NC was used as a control. Lenti-PFKFB3 was amplified by infecting 293T cells (National Collection of Authenticated Cell Cultures, China) for 2 days. Primary mouse chondrocytes were transfected with lentivirus at a multiplicity of infection (MOI) of 250 in the presence of 10 μg/mL polybrene (Beyotime) for 12 h, followed by replacement of medium. Cells were harvested 3 days after transfection.

### Extraction of nuclear and cytoplasmic proteins

Nuclear and cytoplasmic proteins were extracted using the Nuclear and Cytoplasmic Protein Extraction Kit (Beyotime) according to the manufacturer’s protocol.

### SA-β-gal staining

SA-β-Gal staining was performed using the Senescence β-Galactosidase staining kit (Beyotime) as described previously [25]. Briefly, the cells were washed with PBS and incubated with a fixation solution for 15 min at room temperature. Cells were then rinsed and incubated overnight at 37 °C with SA-β-Gal staining solution. Images were captured using an optical microscope (Olympus).

### Comet assay

DNA damage in chondrocytes was evaluated using the Comet Assay Kit (Beyotime) following the manufacturer’s instructions. The comet assay was performed by embedding cells in a layer of low-melting-point agarose over a normal-melting-point agarose slide, followed by cell lysis in cold buffer containing DMSO. After lysis, slides were subjected to alkaline unwinding and electrophoresis at 25 V for 20–30 min. Then, samples were stained with propidium iodide and visualized under a fluorescence microscope (Olympus). Tail moment, calculated as the product of tail length and tail DNA percentage, was used to quantify DNA damage.

### CCK-8

Cell viability was assessed using the CCK-8 assay kit (Beyotime) according to the manufacturer’s instructions.

### Bioinformatic analysis

For bulk sequencing, public transcriptome datasets of OA models from the Gene Expression Omnibus (GEO) database were used in the present study. Transcriptomes of rat OA knees (GSE241794) and human OA cartilage (GSE114007) were used to determine the PFKFB3 expression.

Raw single-cell RNA sequencing (scRNA-seq) data (GSE104782) were downloaded and processed using the Seurat package (v.5.1.0) in R (v.4.4.1).

Interacting networks of PFKFB3 were visualized with Cytoscape v3.9.1 using the top 35 DEGs from PFKFB3-knockdown RNA-seq.

### Statistical analyses

All results were obtained from at least three independent experiments and presented as the means ± s.e.m. Data analysis and graphical presentation were performed with GraphPad Prism 8 (San Diego, USA). A two-tailed Student’s *t*-test was used for comparisons between two groups. For comparisons between more than two groups, one-way analysis of variance (ANOVA) with Tukey’s multiple comparisons was used. P-values less than 0.05 were considered statistically significant.

## Supplementary information

SUPPLEMENTARY MATERIAL

Original data

GSEA analysis

## Data Availability

All data are included in the manuscript or available from the corresponding authors upon reasonable request.
