# Supplementary material for: The glycolytic enzyme PFKFB3 alleviates DNA damage and chondrocyte senescence in osteoarthritis
Source: Cell Death Discov. 2025 Dec 8;12:70. doi: 10.1038/s41420-025-02903-0 (PMC12848158; doi:10.1038/s41420-025-02903-0)
Supplement: Supplementary file 1 — SUPPLEMENTARY MATERIAL [file 41420_2025_2903_MOESM1_ESM.docx]

**The glycolytic enzyme** **PFKFB3 alleviates DNA damage and** **chondrocyte** **senescence in osteoarthritis.**

Bo Liu^1,^^†^, Chenzhong Wang^1,†^, Ziyu Weng^1^, Yi Yang^1^, Yi Shi^2,3^*, Chi Zhang^1,^*

^1^ Department of Orthopedic Surgery, Zhongshan Hospital, Fudan University, Shanghai, China.

^2^ Department of Kidney Transplantation, Zhongshan Hospital, Fudan University, Shanghai, China.

^3^ Shanghai Key Laboratory of Organ Transplantation, Zhongshan Hospital, Fudan University, Shanghai, China.

^†^BL and CW contributed equally to this work.

**This file includes:**

**Supplementary Materials and Methods**

**Supplementary Figures S1-S5**

**Supplementary Tables S1-S3**

**Supplementary Materials and Methods**

**Isolation and Culture of Murine Chondrocytes**

5-7-day-old neonatal C57BL/6 mice were provided by the Department of Laboratory animals, Zhongshan Hospital. Immature murine articular chondrocytes were isolated from the knee joints of neonatal mice according to our previous protocol (1). Primary mouse chondrocytes were cultured in Dulbecco’s Modified Eagle Medium/Nutrient Mixture F-12 (Gibco, Carlsbad, USA) containing 10% fetal bovine serum (Gibco, Carlsbad, USA) in a humidified culture chamber at 37°C with 5% CO2 and 95% air. Primary chondrocytes without passaging were used in the present study. Experiments involving animals were approved by the Ethics Committee for Animal Research of Zhongshan Hospital.

In the present study, three *in vitro* models of DNA damage and cell senescence were used in primary chondrocytes stimulated with 200 μM H_2_O_2_ for three days, 10 μM etoposide for two days, and an ionizing radiation (IR) exposure (10 Gy) for two days.

**Quantitative Real-Time PCR (****qRT-PCR)**

Total RNA was extracted using the TRIzol reagent (Sigma-Aldrich). cDNA was synthesized using the First Strand cDNA synthesis kit (Takara Bio, Shiga, Japan) according to the manufacturer’s instructions. Quantitative PCR analyses were performed using the Hieff qPCR SYBR Green Master Mix (Yeasen, Shanghai, China).

Fluorescence signals were acquired by ABI Quant Studio 7 Flex (Applied Biosystems, Foster City, CA, USA). Gene expression was normalized to the housekeeping gene *β-Actin* and calculated using the 2^-ΔΔC^_T_ method. Primer sequences used for qPCR are listed in Supplementary Table 3.

**Western Blotting**

Western blotting was performed according to standard methods. Briefly, cell lysates were separated by 10% SDS-polyacrylamide gel electrophoresis and transferred to polyvinylidene fluoride membranes. After blocking with 5 % milk (Beyotime), membranes were incubated with primary antibodies. After washing out, the membranes were further incubated with HRP-conjugated secondary antibodies. Protein band signals were detected using chemiluminescence reagents (Beyotime) and visualized using a Tanon Imager 4600 system (Tanon, Shanghai, China). ImageJ software was used to quantify the immunoblots.

**TUNEL Staining**

TUNEL Staining was performed using the One Step TUNEL Apoptosis Assay Kit (C1089; Beyotime) according to the manufacturer’s instructions. Fluorescence signals were obtained using a confocal laser scanning microscope (Olympus).

**RNA Sequencing**

Total RNA from cultured mouse chondrocytes transfected with siPFKFB3 or infected with lenti-PFKFB3 were extracted using TRIzol reagent. The concentration and purity of RNA were assessed using a NanoDrop spectrophotometer (Thermo Fisher Scientific, Waltham, MA, USA). RNA sample (1 μg) was used for the transcriptome library construction by LC-Bio Technology Co., Ltd (Hangzhou, China). The library, with an average insert size of 300 ± 50 bp, was sequenced as 2×150 bp paired-end reads on an Illumina NovaSeq™ 6000 (LC-Bio Technology Co., Ltd., Hangzhou) following standard protocols. Differentially expressed genes (DEGs) were defined as fold change (FC)≥2 and adjusted P value≤0.05. The online platform of LC-Bio Technology Co., Ltd. was used for subsequent analyses.

**Reference**

1. bo, L., Chenzhong, W., Ziyu, W., Yi, Y., Hong, Z., Yueqi, Z., et al. (2023) Glycolytic enzyme PKM2 regulates cell senescence but not inflammation in the process of osteoarthritis.


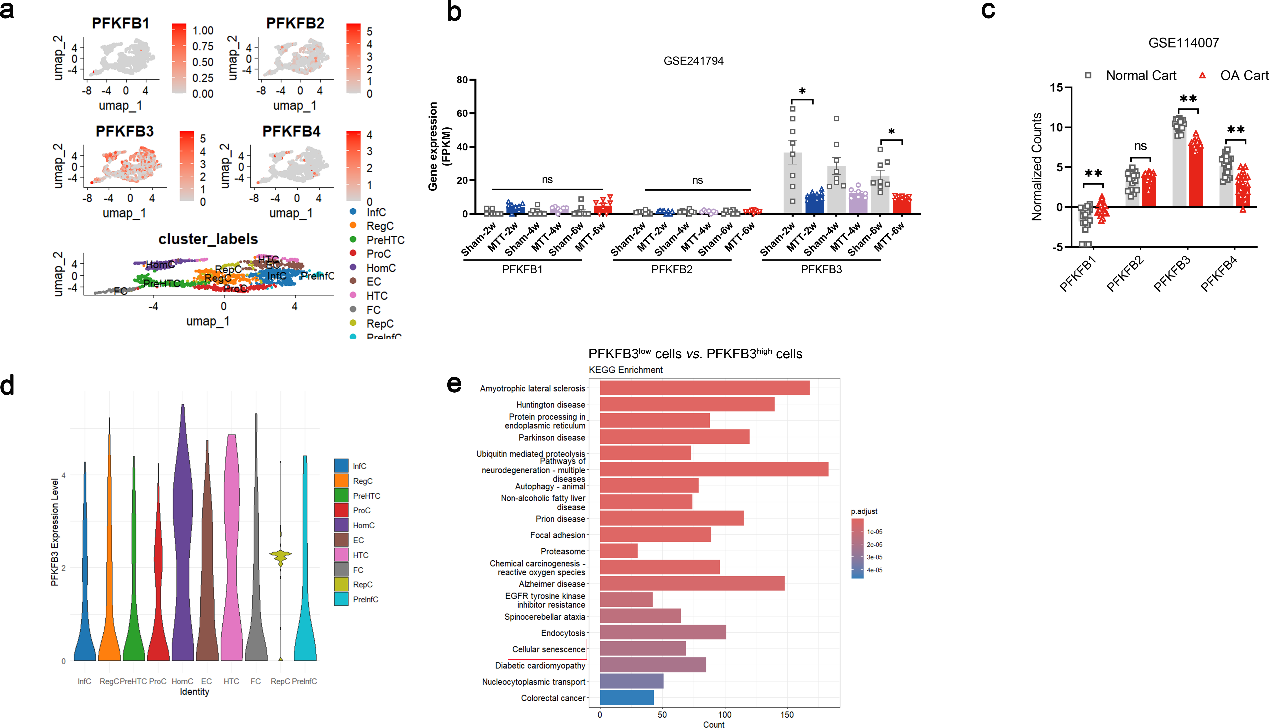
**Supplementary Figures**

**Supplementary Figure 1.** **PFKFB3 expression is associated with OA risk.**

(a) Uniform manifold approximation and projection (UMAP) visualization of single-cell RNA sequencing (scRNA-seq) dataset GSE104782 of human osteoarthritis cartilage (lower panel) and dot plots showing the expression of *PFKFB* family (*PFKFB1-4*) on the UMAP (upper panel). (b) Expression of the *PFKFB1*, *PFKFB2,* and *PFKFB3* genes in cartilage of MMT rats. Data presented as means ± s.e.m., n=8, paired Student's t-test. (c) Expression of the *PFKFB* family (PFKFB1-4) in human non- and OA cartilage tissue (GSE104782). Data presented as means ± s.e.m., n (non-OA Cart)=18, n (OA Cart)=20, unpaired Student's t-test. (d) Violin plots displaying expression of *PFKFB3* in scRNA-seq dataset GSE104782. (e) Kyoto Encyclopedia of Genes and Genomes (KEGG) pathway enrichment analysis for differentially expressed genes between PFKFB3^low^ cells and PFKFB3^high^ cells among HomC subpopulations identified in scRNA-seq dataset GSE114007. *P < 0.05, **P < 0.01. ns, not significant.


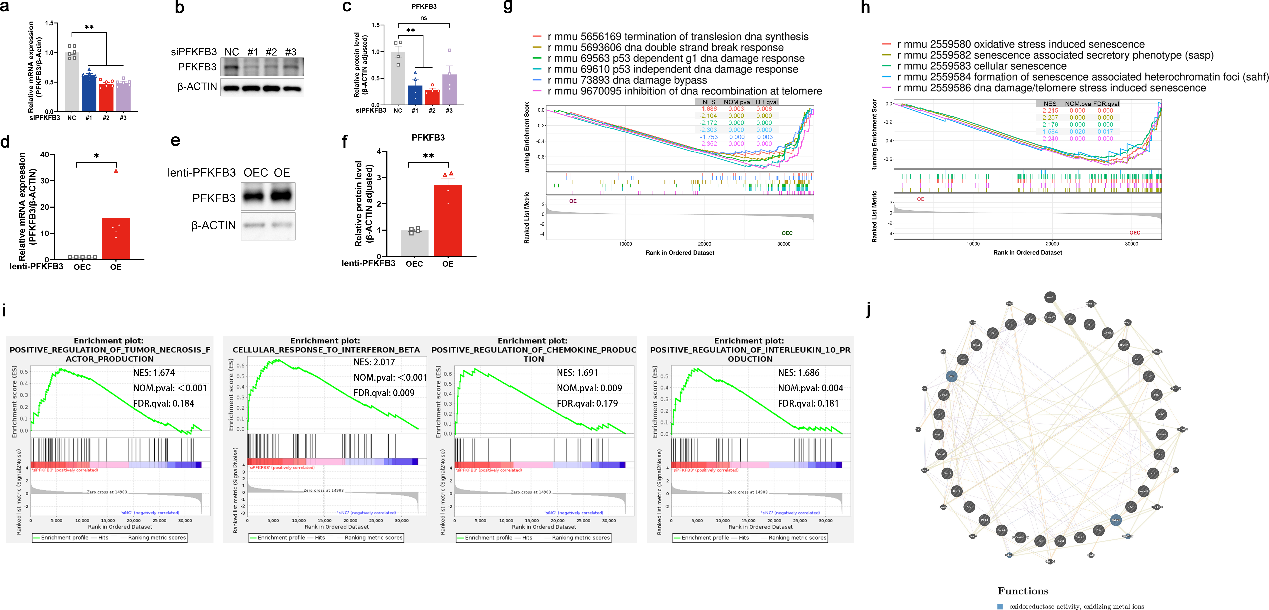
**Supplementary Figure 2. PFKFB3 is associated with DNA damage and senescence in chondrocytes.**

(a) Messenger RNA (mRNA) expression of PFKFB3 in cultured cells transfected with siPFKFB3. Data presented as means ± s.e.m., n=6, one-way ANOVA with Tukey’s comparisons. (b) Representative blots and (c) densitometric quantification of PFKFB3 in cultured cells transfected with siPFKFB3. Data presented as means ± s.e.m., n=4, one-way ANOVA with Tukey’s comparisons. (d) mRNA expression of PFKFB3 in cultured cells infected with lentivirus overexpressing PFKFB3 (lenti-PFKFB3). Data presented as means ± s.e.m., n=5, paired Student's t-test. (e) Representative blots and (f) densitometric quantification of PFKFB3 in cultured cells infected with lenti-PFKFB3. Data presented as means ± s.e.m., n=4, paired Student's t-test. (g) Gene Set Enrichment Analysis (GSEA) associated with DNA damage response and (h) cellular senescence in PFKFB3-overexpressed chondrocytes. (i) GSEA associated with senescence-associated secretory phenotypes (SASPs) in PFKFB3-silenced chondrocytes. (j) Protein-protein interaction network analysis revealed the top 20 PFKFB3 binding proteins based on the Genemania database. *P < 0.05, **P < 0.01. ns, not significant.


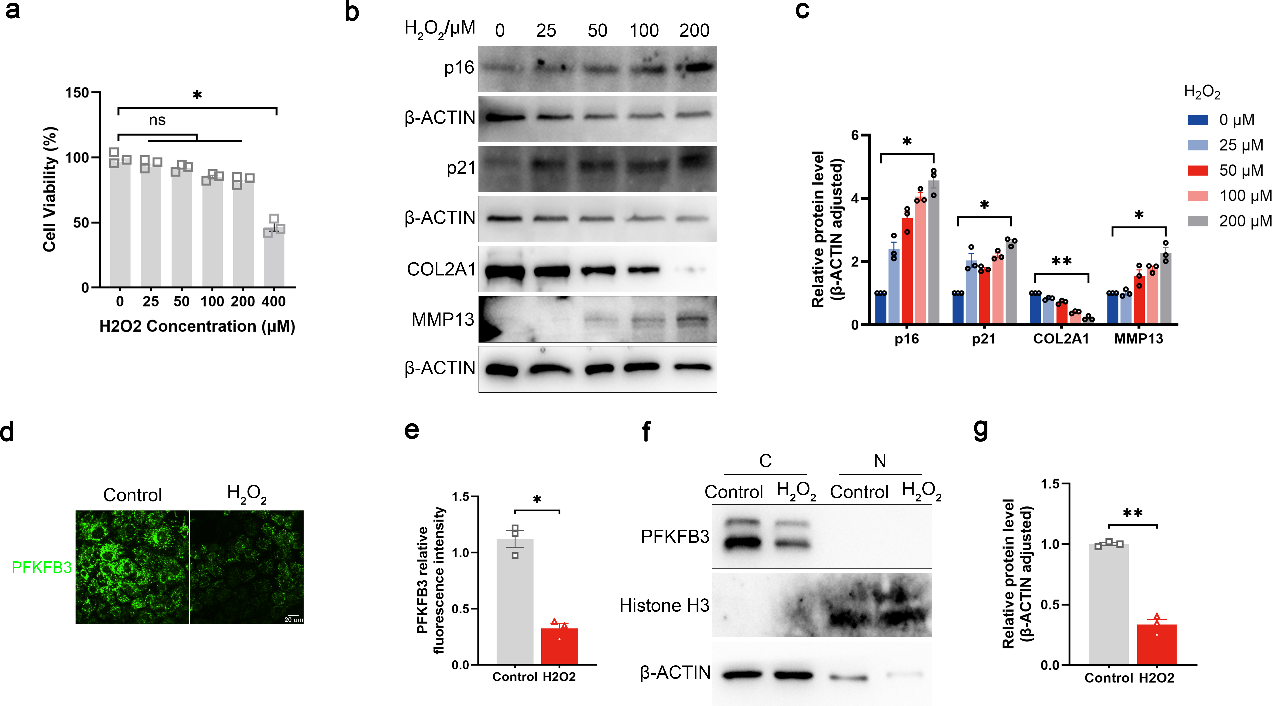
**Supplementary Figure 3. H_2_O_2_ stimulation decreases the PFKFB3 expression in chondrocytes.**

(a) Cell viability assessed by CCK-8 assay after stimulating chondrocytes with H₂O₂ for 72 hours. Data presented as means ± s.e.m., n=3, paired Student's t-test. (b) Representative blots and (c) densitometric quantification of p16, p21, COL2A1, and MMP13 in chondrocytes stimulated with H₂O₂ for 72 hours. Data presented as means ± s.e.m., n=3, one-way ANOVA with Tukey’s comparisons. (d) Immunofluorescence staining and (e) quantification of PFKFB3 signals in chondrocytes stimulated with H_2_O_2_ (200 μM) for 72 hours. Data presented as means ± s.e.m., n=3, paired Student's t-test. (f) Representative blots and (g) densitometric quantification of PFKFB3 in chondrocytes stimulated with H₂O₂ (200 μM) for 72 hours. β-ACTIN and Histone H3 were used as loading controls for the cytoplasmic and nuclear fractions, respectively. Data presented as means ± s.e.m., n=3, paired Student's t-test. ns, not significant. *P < 0.05, **P < 0.01. ns, not significant.

**
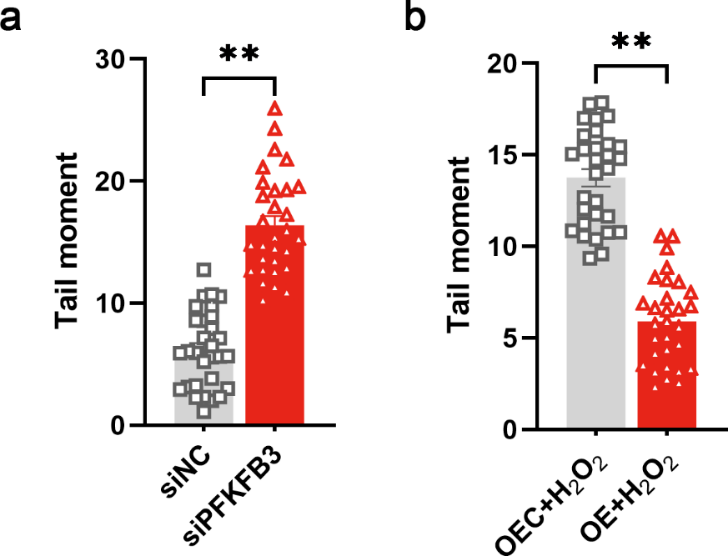
Supplementary Figure 4. PFKFB3 alleviates DNA damage.**

(a) Tailed moment analysis of comet assay in chondrocytes transfected with siPFKFB3. Data presented as means ± s.e.m., n=3 (10 cells randomly selected in each experiment), unpaired Student's t-test. (b) Tailed moment analysis of comet assay in H₂O₂-stimulated chondrocytes infected with lenti-PFKFB3. Data presented as means ± s.e.m., n=3 (10 cells randomly selected in each experiment), unpaired Student's t-test. **P < 0.01.


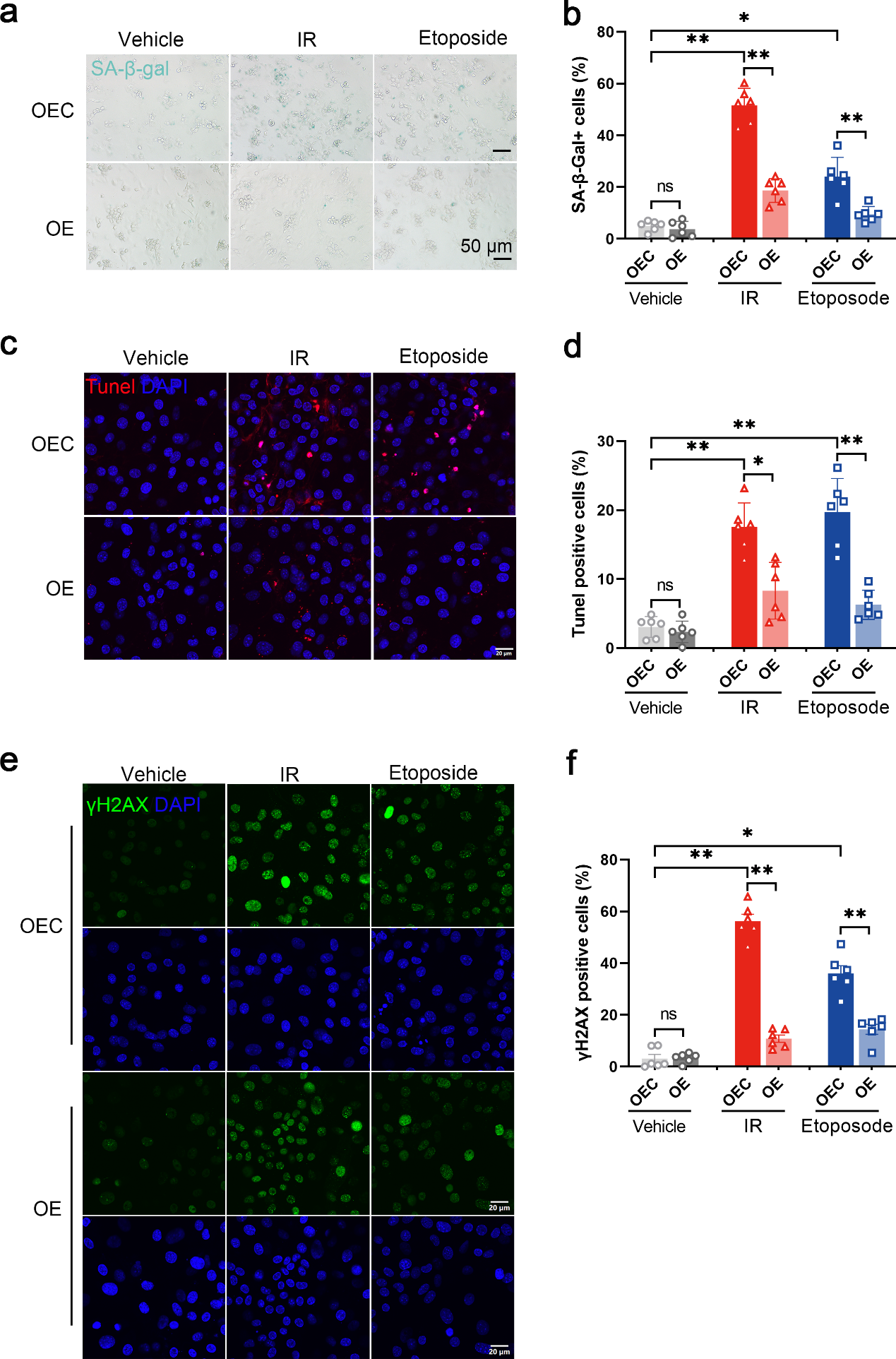
**Supplementary Figure 5. PFKFB3 alleviates IR- and Etoposide-induced DNA damage and senescence in chondrocytes.**

(a) Senescence-associated β-galactosidase (SA-β-Gal) staining and (b) quantification in cultured chondrocytes stimulated with 10 Gy ionizing radiation (IR) or 10 μM etoposide for 48 hours and infected with lenti-PFKFB3. Data presented as means ± s.e.m., n=6, one-way ANOVA with Tukey’s multiple comparisons. (c) Terminal deoxynucleotidyl transferase dUTP nick end labeling staining and (d) quantification in cultured chondrocytes stimulated with 10 Gy IR or 10 μM etoposide for 48 hours and infected with lenti-PFKFB3. Data presented as means ± s.e.m., n=6, one-way ANOVA with Tukey’s multiple comparisons. (e) Immunofluorescence staining and (f) quantification of γH2AX signals in chondrocytes stimulated with 10 Gy IR or 10 μM etoposide for 48 hours and infected with lenti-PFKFB3. The images of OEC and OE groups are the same as those in Figure 4h as these experiments were performed parallelly with shared control groups. Data presented as means ± s.e.m., n=6, one-way ANOVA with Tukey’s multiple comparisons. *P < 0.05, **P < 0.01. ns, not significant.

**Supplementary Figure 6.**
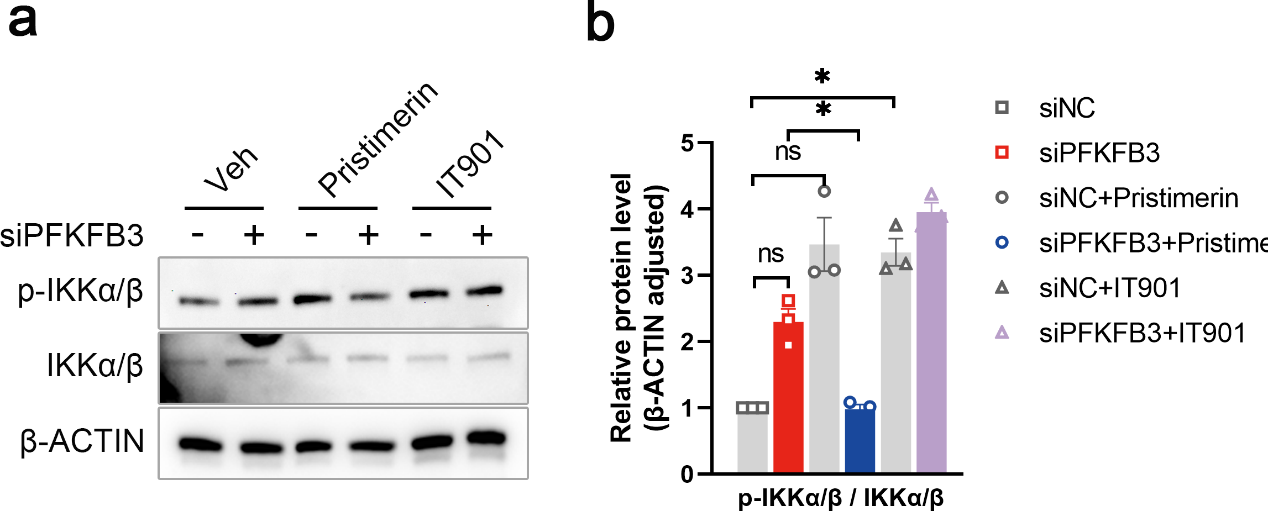
 **PFKFB3 silencing does not modulate p-Ikkα/β expression in chondrocytes.**

(a) Representative blots and (b) densitometric quantification of Ikkα/β and p-Ikkα/β in cultured cells transfected with siPFKFB3 in the presence of NF-κB inhibitors, pristimerin and IT901. Data presented as means ± s.e.m., n=3, one-way ANOVA with Tukey’s multiple comparisons.

**Supplementary Tables. Supplementary Table 1. Clinical characteristics of patients**

| Patient | Gender | Age (years) | Weight (Kg) | ICRS grade* |
| --- | --- | --- | --- | --- |

| 1 | Female | 65 | 68 | II |
| --- | --- | --- | --- | --- |
| 2 | Male | 72 | 75 | IV |
| 3 | Female | 58 | 65 | II |
| 4 | Female | 69 | 55 | III |
| 5 | Male | 54 | 70 | IV |
| 6 | Female | 60 | 72 | III |
| 7 | Female | 43 | 80 | 0 |
| 8 | Male | 58 | 70 | 0 |
| 9 | Male | 43 | 67 | 0 |
| 10 | Male | 55 | 72 | 0 |
| 11 | Male | 37 | 69 | I |
| 12 | Female | 62 | 65 | I |

*ICRS: International Cartilage Repair Society

| **Supplementary Table 2. siRNA information (Mouse)** | |
| --- | --- |
| Gene | Sequence (5′→ 3′) |
| scramble siNC | Sense: UUCUCCGAACGUGUCACGUTT  Antisense: ACGUGACACGUUCGGAGAATT |
| siPFKFB3 #1 | Sense: GGAGUCCAUUUACUUGAAUTT  Antisense: AUUCAAGUAAAUGGACUCCTT |
| siPFKFB3 #2 | Sense: GAGCCUGUGAUCAUGGAAUTT  Antisense: AUUCCAUGAUCACAGGCUCTT |
| siPFKFB3 #3 | Sense: GGACUUGUCACUCAUCAAATT  Antisense: UUUGAUGAGUGACAAGUCCTT |

| **Supplementary Table 3. Primer sequences used in qRT-PCR (Mouse)** | |
| --- | --- |
| Gene | Primer (5′→ 3′) |
| PFKFB3 | Forward primer: CCCAGAGCCGGGTACAGAA  Reverse primer: GGGGAGTTGGTCAGCTTCG |
| β-ACTIN | Forward primer: GGCTGTATTCCCCTCCATCG  Reverse primer: CCAGTTGGTAACAATGCCATGT |
| p16^INK4a^ | Forward primer: GTACCCCGATTCAGGTGAT  Reverse primer: TTGAGCAGAAGAGCTGCTACGT |
| IL-1β | Forward primer: TTCAGGCAGGCAGTATCACTC  Reverse primer: GAAGGTCCACGGGAAAGACAC |
| IL-6 | Forward primer: TAGTCCTTCCTACCCCAATTTCC  Reverse primer: TTGGTCCTTAGCCACTCCTTC |
| IL-10 | Forward primer: CTTACTGACTGGCATGAGGATCA  Reverse primer: GCAGCTCTAGGAGCATGTGG |
| TNF-α | Forward primer: CAGGCGGTGCCTATGTCTC  Reverse primer: CGATCACCCCGAAGTTCAGTAG |
