# Supplementary figures and images for: The glycolytic enzyme PFKFB3 alleviates DNA damage and chondrocyte senescence in osteoarthritis
Source: Cell Death Discov. 2025 Dec 8;12:70. doi: 10.1038/s41420-025-02903-0 (PMC12848158; doi:10.1038/s41420-025-02903-0)

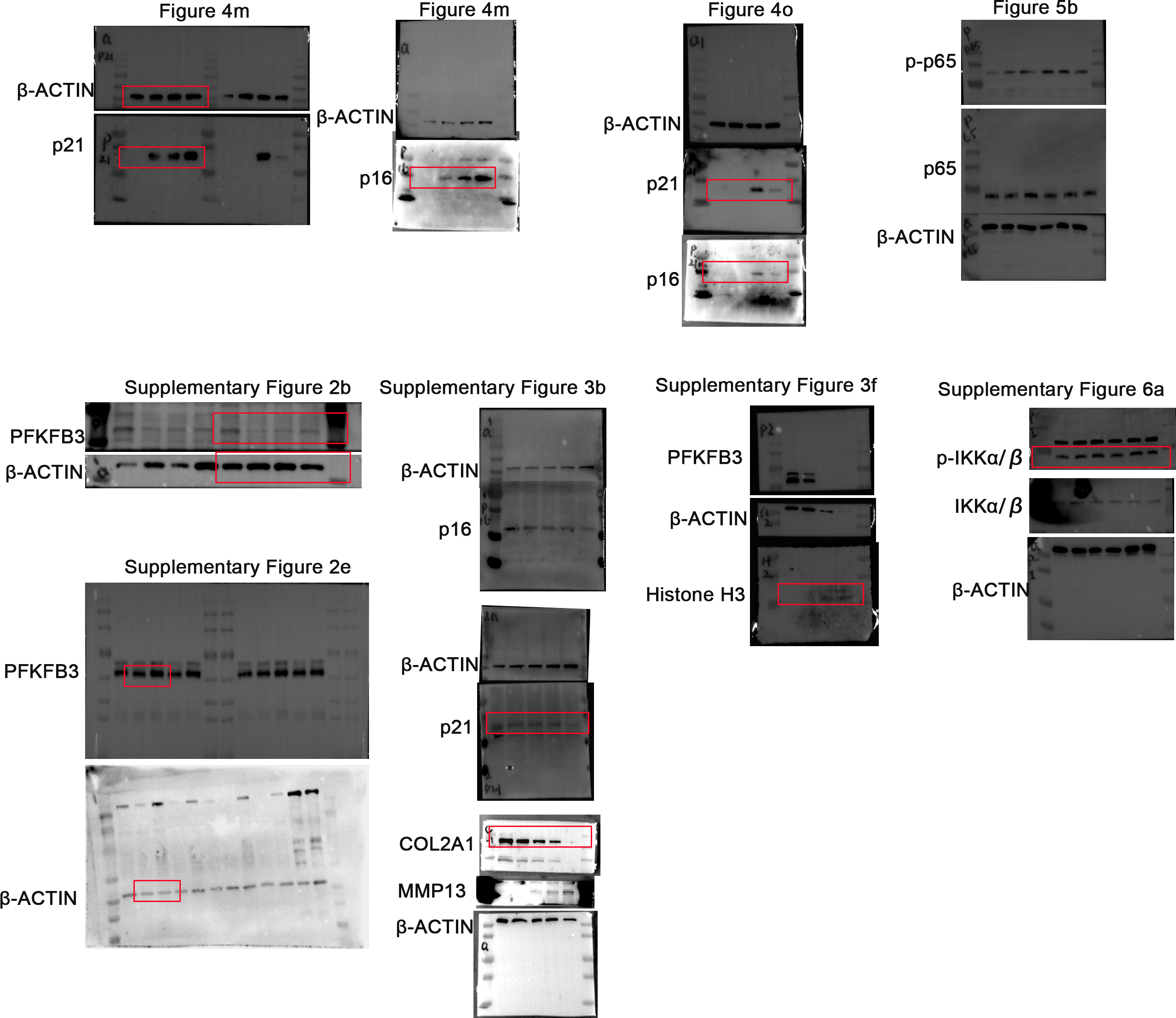

Supplement: Supplementary file 2 — Original data [file 41420_2025_2903_MOESM2_ESM.png]
